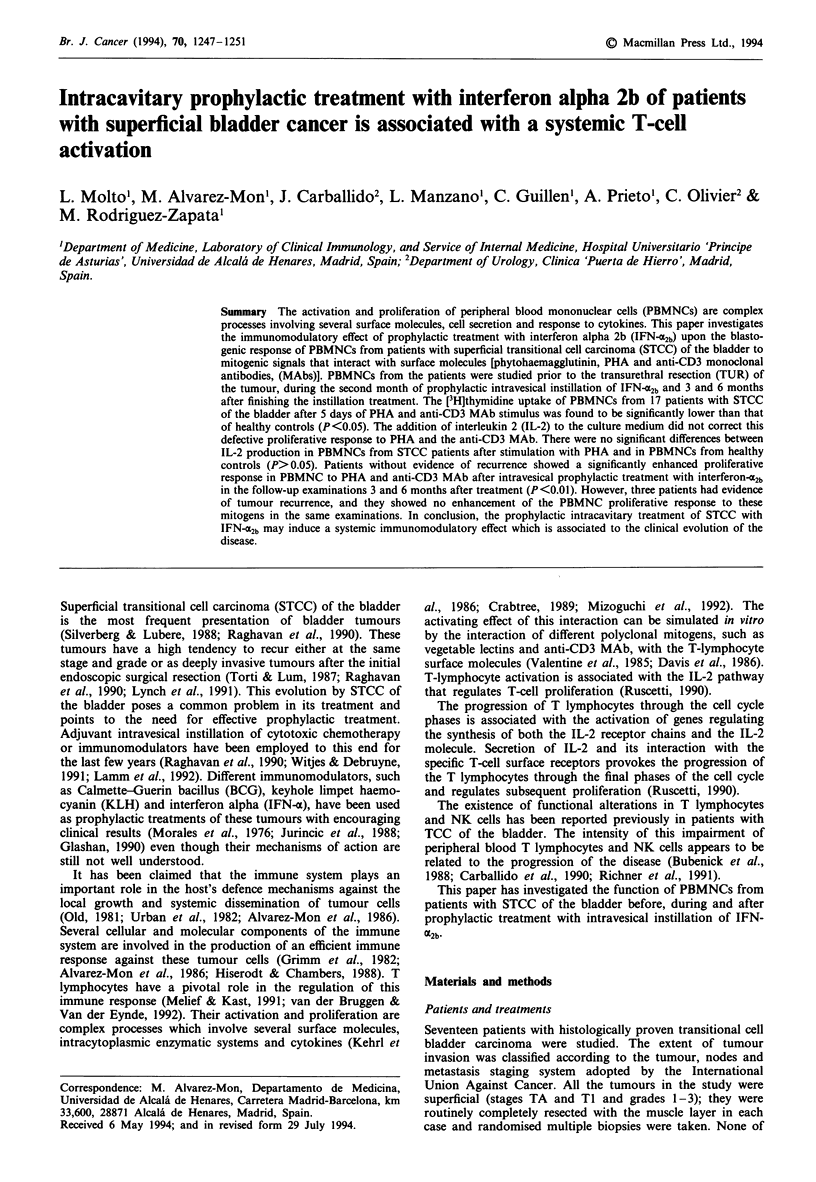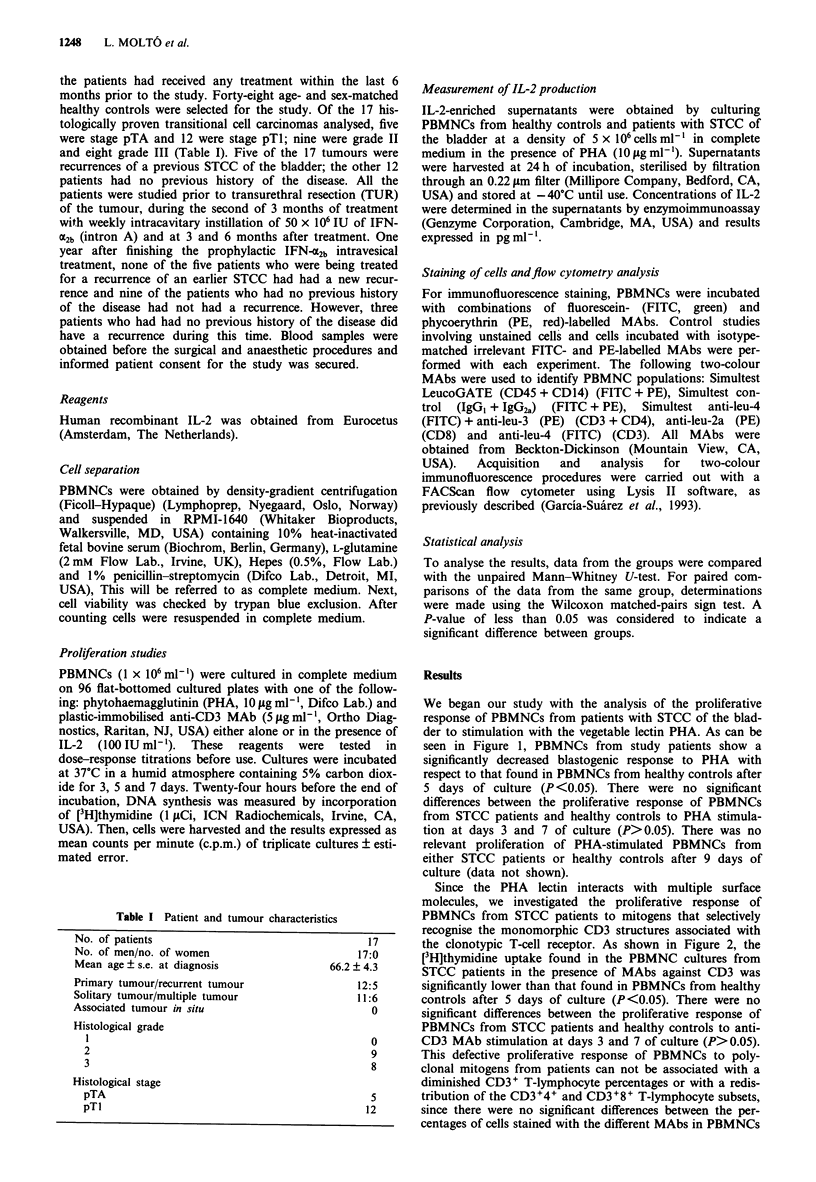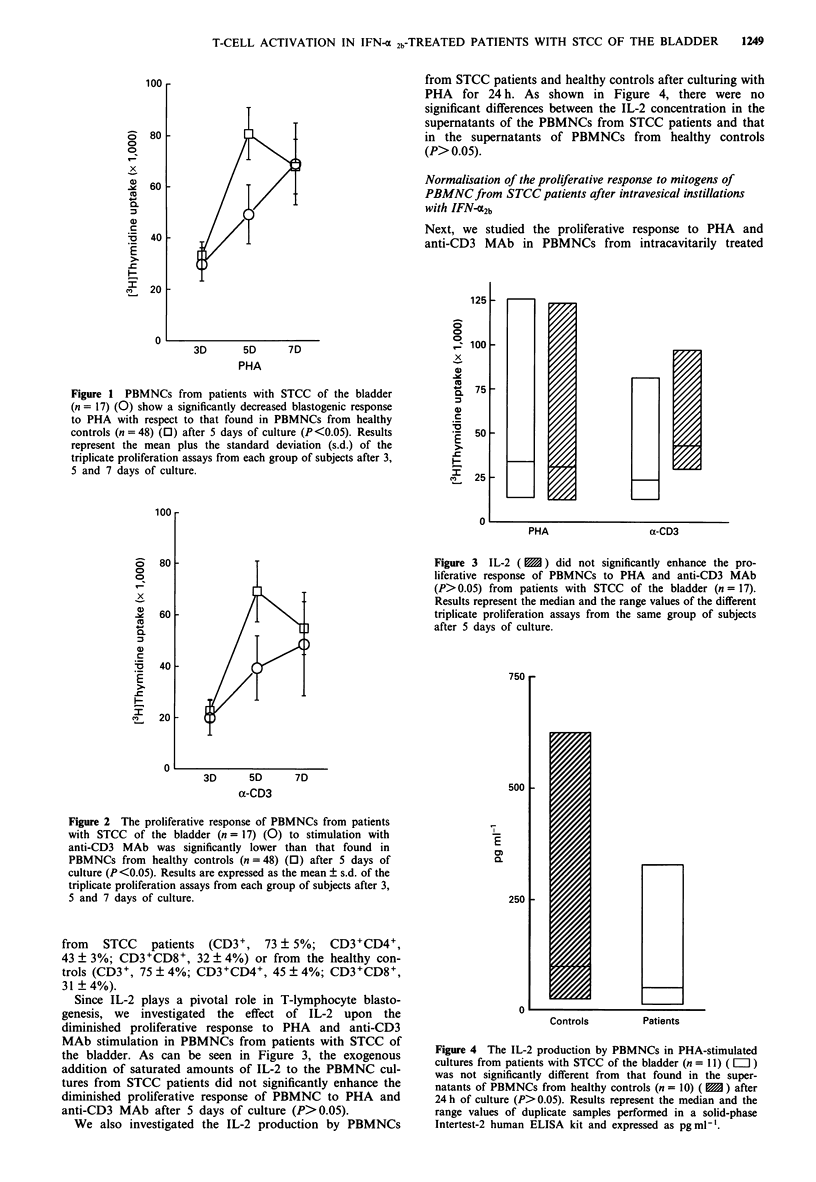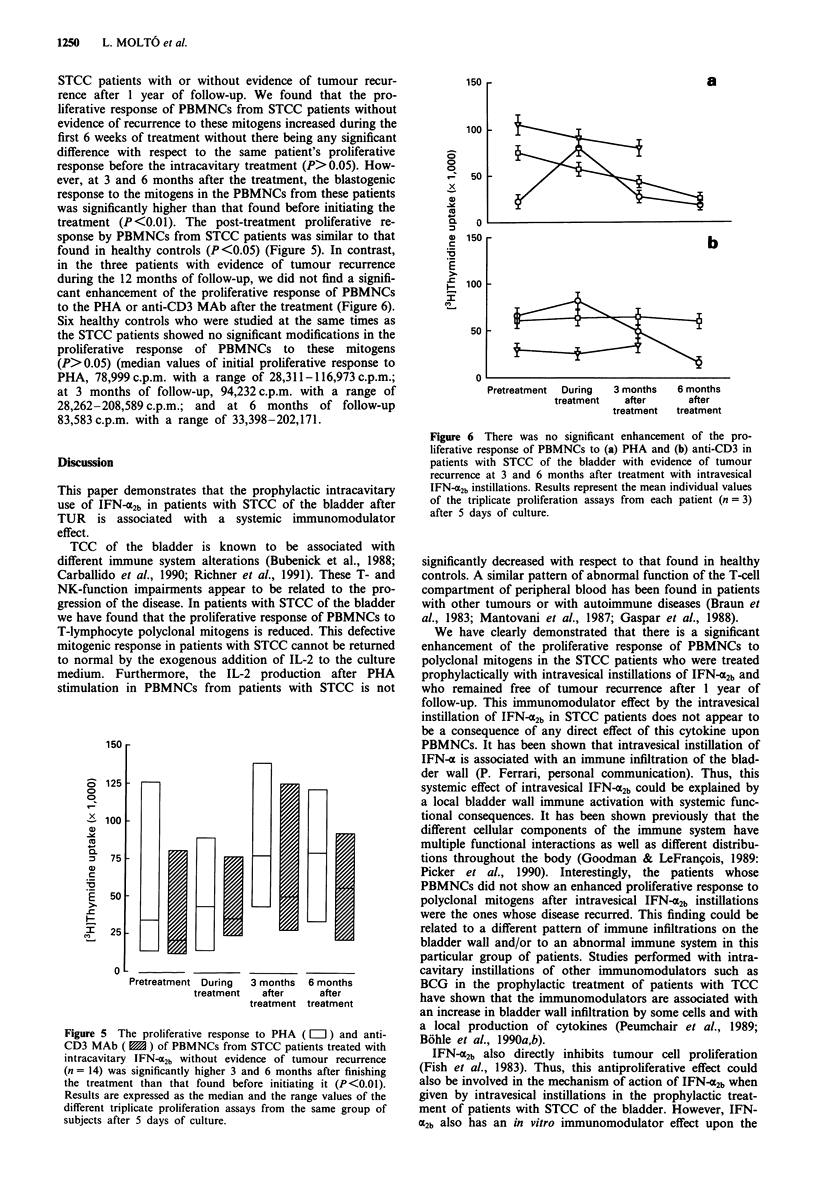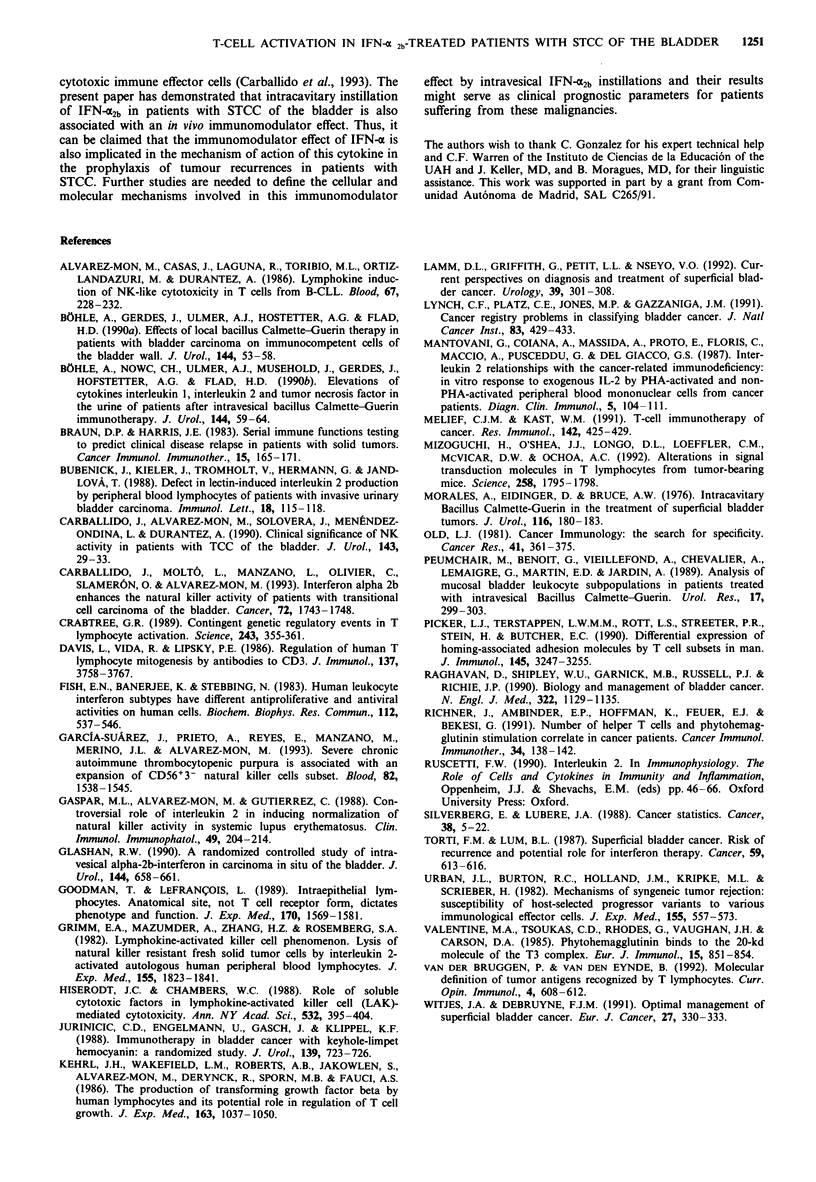# Intracavitary prophylactic treatment with interferon alpha 2b of patients with superficial bladder cancer is associated with a systemic T-cell activation.

**DOI:** 10.1038/bjc.1994.481

**Published:** 1994-12

**Authors:** L. Molto, M. Alvarez-Mon, J. Carballido, L. Manzano, C. Guillen, A. Prieto, C. Olivier, M. Rodriguez-Zapata

**Affiliations:** Department of Medicine, Hospital Universitario Principe de Asturias, Universidad de Alcalá de Henares, Madrid, Spain.

## Abstract

The activation and proliferation of peripheral blood mononuclear cells (PBMNCs) are complex processes involving several surface molecules, cell secretion and response to cytokines. This paper investigates the immunomodulatory effect of prophylactic treatment with interferon alpha 2b (IFN-alpha 2b) upon the blastogenic response of PBMNCs from patients with superficial transitional cell carcinoma (STCC) of the bladder to mitogenic signals that interact with surface molecules [phytohaemagglutinin, PHA and anti-CD3 monoclonal antibodies, (MAbs)]. PBMNCs from the patients were studied prior to the transurethral resection (TUR) of the tumour, during the second month of prophylactic intravesical instillation of IFN-alpha 2b and 3 and 6 months after finishing the instillation treatment. The [3H]thymidine uptake of PBMNCs from 17 patients with STCC of the bladder after 5 days of PHA and anti-CD3 MAb stimulus was found to be significantly lower than that of healthy controls (P < 0.05). The addition of interleukin 2 (IL-2) to the culture medium did not correct this defective proliferative response to PHA and the anti-CD3 MAb. There were no significant differences between IL-2 production in PBMNCs from STCC patients after stimulation with PHA and in PBMNCs from healthy controls (P > 0.05). Patients without evidence of recurrence showed a significantly enhanced proliferative response in PBMNC to PHA and anti-CD3 MAb after intravesical prophylactic treatment with interferon-alpha 2b in the follow-up examinations 3 and 6 months after treatment (P < 0.01). However, three patients had evidence of tumour recurrence, and they showed no enhancement of the PBMNC proliferative response to these mitogens in the same examinations. In conclusion, the prophylactic intracavitary treatment of STCC with IFN-alpha 2b may induce a systemic immunomodulatory effect which is associated to the clinical evolution of the disease.


Br. J. Cancer (1994), 70, 1247–1251 © Macmillan Press Ltd., 1994

### Intracavitary prophylactic treatment with interferon alpha 2b of patients with superficial bladder cancer is associated with a systemic T-cell activation

L. Molto[1], M. Alvarez-Mon[1], J. Carballido[2], L. Manzano[1], C. Guillen[1], A. Prieto[1], C. Olivier[2] & M. Rodriguez-Zapata[1]

[1]*Department of Medicine, Laboratory of Clinical Immunology, and Service of Internal Medicine, Hospital Universitario 'Principe de Asturias', Universidad de Alcalá de Henares, Madrid, Spain;* [2]*Department of Urology, Clinica 'Puerta de Hierro', Madrid, Spain.*

**Summary** The activation and proliferation of peripheral blood mononuclear cells (PBMNCs) are complex processes involving several surface molecules, cell secretion and response to cytokines. This paper investigates the immunomodulatory effect of prophylactic treatment with interferon alpha 2b (IFN-$\alpha_{2b}$) upon the blastogenic response of PBMNCs from patients with superficial transitional cell carcinoma (STCC) of the bladder to mitogenic signals that interact with surface molecules [phytohaemagglutinin, PHA and anti-CD3 monoclonal antibodies, (MAbs)]. PBMNCs from the patients were studied prior to the transurethral resection (TUR) of the tumour, during the second month of prophylactic intravesical instillation of IFN-$\alpha_{2b}$ and 3 and 6 months after finishing the instillation treatment. The [$^3$H]thymidine uptake of PBMNCs from 17 patients with STCC of the bladder after 5 days of PHA and anti-CD3 MAb stimulus was found to be significantly lower than that of healthy controls ($P<0.05$). The addition of interleukin 2 (IL-2) to the culture medium did not correct this defective proliferative response to PHA and the anti-CD3 MAb. There were no significant differences between IL-2 production in PBMNCs from STCC patients after stimulation with PHA and in PBMNCs from healthy controls ($P>0.05$). Patients without evidence of recurrence showed a significantly enhanced proliferative response in PBMNC to PHA and anti-CD3 MAb after intravesical prophylactic treatment with interferon-$\alpha_{2b}$ in the follow-up examinations 3 and 6 months after treatment ($P<0.01$). However, three patients had evidence of tumour recurrence, and they showed no enhancement of the PBMNC proliferative response to these mitogens in the same examinations. In conclusion, the prophylactic intracavitary treatment of STCC with IFN-$\alpha_{2b}$ may induce a systemic immunomodulatory effect which is associated to the clinical evolution of the disease.

Superficial transitional cell carcinoma (STCC) of the bladder is the most frequent presentation of bladder tumours (Silverberg & Lubere, 1988; Raghavan *et al.*, 1990). These tumours have a high tendency to recur either at the same stage and grade or as deeply invasive tumours after the initial endoscopic surgical resection (Torti & Lum, 1987; Raghavan *et al.*, 1990; Lynch *et al.*, 1991). This evolution by STCC of the bladder poses a common problem in its treatment and points to the need for effective prophylactic treatment. Adjuvant intravesical instillation of cytotoxic chemotherapy or immunomodulators have been employed to this end for the last few years (Raghavan *et al.*, 1990; Witjes & Debruyne, 1991; Lamm *et al.*, 1992). Different immunomodulators, such as Calmette–Guerin bacillus (BCG), keyhole limpet haemocyanin (KLH) and interferon alpha (IFN-α), have been used as prophylactic treatments of these tumours with encouraging clinical results (Morales *et al.*, 1976; Jurincic *et al.*, 1988; Glashan, 1990) even though their mechanisms of action are still not well understood.

It has been claimed that the immune system plays an important role in the host's defence mechanisms against the local growth and systemic dissemination of tumour cells (Old, 1981; Urban *et al.*, 1982; Alvarez-Mon *et al.*, 1986). Several cellular and molecular components of the immune system are involved in the production of an efficient immune response against these tumour cells (Grimm *et al.*, 1982; Alvarez-Mon *et al.*, 1986; Hiserodt & Chambers, 1988). T lymphocytes have a pivotal role in the regulation of this immune response (Melief & Kast, 1991; van der Bruggen & Van der Eynde, 1992). Their activation and proliferation are complex processes which involve several surface molecules, intracytoplasmic enzymatic systems and cytokines (Kehrl *et al.*, 1986; Crabtree, 1989; Mizoguchi *et al.*, 1992). The activating effect of this interaction can be simulated *in vitro* by the interaction of different polyclonal mitogens, such as vegetable lectins and anti-CD3 MAb, with the T-lymphocyte surface molecules (Valentine *et al.*, 1985; Davis *et al.*, 1986). T-lymphocyte activation is associated with the IL-2 pathway that regulates T-cell proliferation (Ruscetti, 1990).

The progression of T lymphocytes through the cell cycle phases is associated with the activation of genes regulating the synthesis of both the IL-2 receptor chains and the IL-2 molecule. Secretion of IL-2 and its interaction with the specific T-cell surface receptors provokes the progression of the T lymphocytes through the final phases of the cell cycle and regulates subsequent proliferation (Ruscetti, 1990).

The existence of functional alterations in T lymphocytes and NK cells has been reported previously in patients with TCC of the bladder. The intensity of this impairment of peripheral blood T lymphocytes and NK cells appears to be related to the progression of the disease (Bubenick *et al.*, 1988; Carballido *et al.*, 1990; Richner *et al.*, 1991).

This paper has investigated the function of PBMNCs from patients with STCC of the bladder before, during and after prophylactic treatment with intravesical instillation of IFN-$\alpha_{2b}$.

### Materials and methods

### *Patients and treatments*

Seventeen patients with histologically proven transitional cell bladder carcinoma were studied. The extent of tumour invasion was classified according to the tumour, nodes and metastasis staging system adopted by the International Union Against Cancer. All the tumours in the study were superficial (stages TA and T1 and grades 1–3); they were routinely completely resected with the muscle layer in each case and randomised multiple biopsies were taken. None of

Correspondence: M. Alvarez-Mon, Departamento de Medicina, Universidad de Alcalá de Henares, Carretera Madrid-Barcelona, km 33,600, 28871 Alcalá de Henares, Madrid, Spain.
Received 6 May 1994; and in revised form 29 July 1994.

the patients had received any treatment within the last 6 months prior to the study. Forty-eight age- and sex-matched healthy controls were selected for the study. Of the 17 histologically proven transitional cell carcinomas analysed, five were stage pTA and 12 were stage pT1; nine were grade II and eight grade III (Table I). Five of the 17 tumours were recurrences of a previous STCC of the bladder; the other 12 patients had no previous history of the disease. All the patients were studied prior to transurethral resection (TUR) of the tumour, during the second of 3 months of treatment with weekly intracavitary instillation of $50 \times 10^6$ IU of IFN-$\alpha_{2b}$ (intron A) and at 3 and 6 months after treatment. One year after finishing the prophylactic IFN-$\alpha_{2b}$ intravesical treatment, none of the five patients who were being treated for a recurrence of an earlier STCC had had a new recurrence and nine of the patients who had no previous history of the disease had not had a recurrence. However, three patients who had had no previous history of the disease did have a recurrence during this time. Blood samples were obtained before the surgical and anaesthetic procedures and informed patient consent for the study was secured.

*Reagents*

Human recombinant IL-2 was obtained from Eurocetus (Amsterdam, The Netherlands).

*Cell separation*

PBMNCs were obtained by density-gradient centrifugation (Ficoll–Hypaque) (Lymphoprep, Nyegaard, Oslo, Norway) and suspended in RPMI-1640 (Whitaker Bioproducts, Walkersville, MD, USA) containing 10% heat-inactivated fetal bovine serum (Biochrom, Berlin, Germany), L-glutamine (2 mM Flow Lab., Irvine, UK), Hepes (0.5%, Flow Lab.) and 1% penicillin–streptomycin (Difco Lab., Detroit, MI, USA), This will be referred to as complete medium. Next, cell viability was checked by trypan blue exclusion. After counting cells were resuspended in complete medium.

*Proliferation studies*

PBMNCs ($1 \times 10^6$ ml$^{-1}$) were cultured in complete medium on 96 flat-bottomed cultured plates with one of the following: phytohaemagglutinin (PHA, 10 μg ml$^{-1}$, Difco Lab.) and plastic-immobilised anti-CD3 MAb (5 μg ml$^{-1}$, Ortho Diagnostics, Raritan, NJ, USA) either alone or in the presence of IL-2 (100 IU ml$^{-1}$). These reagents were tested in dose–response titrations before use. Cultures were incubated at 37°C in a humid atmosphere containing 5% carbon dioxide for 3, 5 and 7 days. Twenty-four hours before the end of incubation, DNA synthesis was measured by incorporation of [$^3$H]thymidine (1 μCi, ICN Radiochemicals, Irvine, CA, USA). Then, cells were harvested and the results expressed as mean counts per minute (c.p.m.) of triplicate cultures ± estimated error.

**Table I** Patient and tumour characteristics

| | |
|---|---|
| No. of patients | 17 |
| No. of men/no. of women | 17:0 |
| Mean age ± s.e. at diagnosis | 66.2 ± 4.3 |
| Primary tumour/recurrent tumour | 12:5 |
| Solitary tumour/multiple tumour | 11:6 |
| Associated tumour *in situ* | 0 |
| Histological grade | |
| 1 | 0 |
| 2 | 9 |
| 3 | 8 |
| Histological stage | |
| pTA | 5 |
| pT1 | 12 |

*Measurement of IL-2 production*

IL-2-enriched supernatants were obtained by culturing PBMNCs from healthy controls and patients with STCC of the bladder at a density of $5 \times 10^6$ cells ml$^{-1}$ in complete medium in the presence of PHA (10 μg ml$^{-1}$). Supernatants were harvested at 24 h of incubation, sterilised by filtration through an 0.22 μm filter (Millipore Company, Bedford, CA, USA) and stored at −40°C until use. Concentrations of IL-2 were determined in the supernatants by enzymoimmunoassay (Genzyme Corporation, Cambridge, MA, USA) and results expressed in pg ml$^{-1}$.

*Staining of cells and flow cytometry analysis*

For immunofluorescence staining, PBMNCs were incubated with combinations of fluorescein- (FITC, green) and phycoerythrin (PE, red)-labelled MAbs. Control studies involving unstained cells and cells incubated with isotype-matched irrelevant FITC- and PE-labelled MAbs were performed with each experiment. The following two-colour MAbs were used to identify PBMNC populations: Simultest LeucoGATE (CD45 + CD14) (FITC + PE), Simultest control ($IgG_1 + IgG_{2a}$) (FITC + PE), Simultest anti-leu-4 (FITC) + anti-leu-3 (PE) (CD3 + CD4), anti-leu-2a (PE) (CD8) and anti-leu-4 (FITC) (CD3). All MAbs were obtained from Beckton-Dickinson (Mountain View, CA, USA). Acquisition and analysis for two-colour immunofluorescence procedures were carried out with a FACScan flow cytometer using Lysis II software, as previously described (García-Suárez *et al*., 1993).

*Statistical analysis*

To analyse the results, data from the groups were compared with the unpaired Mann–Whitney *U*-test. For paired comparisons of the data from the same group, determinations were made using the Wilcoxon matched-pairs sign test. A *P*-value of less than 0.05 was considered to indicate a significant difference between groups.

### Results

We began our study with the analysis of the proliferative response of PBMNCs from patients with STCC of the bladder to stimulation with the vegetable lectin PHA. As can be seen in Figure 1, PBMNCs from study patients show a significantly decreased blastogenic response to PHA with respect to that found in PBMNCs from healthy controls after 5 days of culture ($P < 0.05$). There were no significant differences between the proliferative response of PBMNCs from STCC patients and healthy controls to PHA stimulation at days 3 and 7 of culture ($P > 0.05$). There was no relevant proliferation of PHA-stimulated PBMNCs from either STCC patients or healthy controls after 9 days of culture (data not shown).

Since the PHA lectin interacts with multiple surface molecules, we investigated the proliferative response of PBMNCs from STCC patients to mitogens that selectively recognise the monomorphic CD3 structures associated with the clonotypic T-cell receptor. As shown in Figure 2, the [$^3$H]thymidine uptake found in the PBMNC cultures from STCC patients in the presence of MAbs against CD3 was significantly lower than that found in PBMNCs from healthy controls after 5 days of culture ($P < 0.05$). There were no significant differences between the proliferative response of PBMNCs from STCC patients and healthy controls to anti-CD3 MAb stimulation at days 3 and 7 of culture ($P > 0.05$). This defective proliferative response of PBMNCs to polyclonal mitogens from patients can not be associated with a diminished $CD3^+$ T-lymphocyte percentages or with a redistribution of the $CD3^+4^+$ and $CD3^+8^+$ T-lymphocyte subsets, since there were no significant differences between the percentages of cells stained with the different MAbs in PBMNCs

**Figure 1** PBMNCs from patients with STCC of the bladder ($n = 17$) (O) show a significantly decreased blastogenic response to PHA with respect to that found in PBMNCs from healthy controls ($n = 48$) (□) after 5 days of culture ($P<0.05$). Results represent the mean plus the standard deviation (s.d.) of the triplicate proliferation assays from each group of subjects after 3, 5 and 7 days of culture.

**Figure 2** The proliferative response of PBMNCs from patients with STCC of the bladder ($n = 17$) (O) to stimulation with anti-CD3 MAb was significantly lower than that found in PBMNCs from healthy controls ($n = 48$) (□) after 5 days of culture ($P<0.05$). Results are expressed as the mean ± s.d. of the triplicate proliferation assays from each group of subjects after 3, 5 and 7 days of culture.

from STCC patients ($CD3^+$, 73 ± 5%; $CD3^+CD4^+$, 43 ± 3%; $CD3^+CD8^+$, 32 ± 4%) or from the healthy controls ($CD3^+$, 75 ± 4%; $CD3^+CD4^+$, 45 ± 4%; $CD3^+CD8^+$, 31 ± 4%).

Since IL-2 plays a pivotal role in T-lymphocyte blastogenesis, we investigated the effect of IL-2 upon the diminished proliferative response to PHA and anti-CD3 MAb stimulation in PBMNCs from patients with STCC of the bladder. As can be seen in Figure 3, the exogenous addition of saturated amounts of IL-2 to the PBMNC cultures from STCC patients did not significantly enhance the diminished proliferative response of PBMNC to PHA and anti-CD3 MAb after 5 days of culture ($P>0.05$).

We also investigated the IL-2 production by PBMNCs from STCC patients and healthy controls after culturing with PHA for 24 h. As shown in Figure 4, there were no significant differences between the IL-2 concentration in the supernatants of the PBMNCs from STCC patients and that in the supernatants of PBMNCs from healthy controls ($P>0.05$).

*Normalisation of the proliferative response to mitogens of PBMNC from STCC patients after intravesical instillations with IFN-$\alpha_{2b}$*

Next, we studied the proliferative response to PHA and anti-CD3 MAb in PBMNCs from intracavitarily treated

**Figure 3** IL-2 (▨) did not significantly enhance the proliferative response of PBMNCs to PHA and anti-CD3 MAb ($P>0.05$) from patients with STCC of the bladder ($n = 17$). Results represent the median and the range values of the different triplicate proliferation assays from the same group of subjects after 5 days of culture.

**Figure 4** The IL-2 production by PBMNCs in PHA-stimulated cultures from patients with STCC of the bladder ($n = 11$) (□) was not significantly different from that found in the supernatants of PBMNCs from healthy controls ($n = 10$) (▨) after 24 h of culture ($P>0.05$). Results represent the median and the range values of duplicate samples performed in a solid-phase Intertest-2 human ELISA kit and expressed as pg ml$^{-1}$.

STCC patients with or without evidence of tumour recurrence after 1 year of follow-up. We found that the proliferative response of PBMNCs from STCC patients without evidence of recurrence to these mitogens increased during the first 6 weeks of treatment without there being any significant difference with respect to the same patient's proliferative response before the intracavitary treatment ($P > 0.05$). However, at 3 and 6 months after the treatment, the blastogenic response to the mitogens in the PBMNCs from these patients was significantly higher than that found before initiating the treatment ($P < 0.01$). The post-treatment proliferative response by PBMNCs from STCC patients was similar to that found in healthy controls ($P < 0.05$) (Figure 5). In contrast, in the three patients with evidence of tumour recurrence during the 12 months of follow-up, we did not find a significant enhancement of the proliferative response of PBMNCs to the PHA or anti-CD3 MAb after the treatment (Figure 6). Six healthy controls who were studied at the same times as the STCC patients showed no significant modifications in the proliferative response of PBMNCs to these mitogens ($P > 0.05$) (median values of initial proliferative response to PHA, 78,999 c.p.m. with a range of 28,311–116,973 c.p.m.; at 3 months of follow-up, 94,232 c.p.m. with a range of 28,262–208,589 c.p.m.; and at 6 months of follow-up 83,583 c.p.m. with a range of 33,398–202,171.

### Discussion

This paper demonstrates that the prophylactic intracavitary use of IFN-$\alpha_{2b}$ in patients with STCC of the bladder after TUR is associated with a systemic immunomodulator effect.

TCC of the bladder is known to be associated with different immune system alterations (Bubenick et al., 1988; Carballido *et al.*, 1990; Richner *et al.*, 1991). These T- and NK-function impairments appear to be related to the progression of the disease. In patients with STCC of the bladder we have found that the proliferative response of PBMNCs to T-lymphocyte polyclonal mitogens is reduced. This defective mitogenic response in patients with STCC cannot be returned to normal by the exogenous addition of IL-2 to the culture medium. Furthermore, the IL-2 production after PHA stimulation in PBMNCs from patients with STCC is not significantly decreased with respect to that found in healthy controls. A similar pattern of abnormal function of the T-cell compartment of peripheral blood has been found in patients with other tumours or with autoimmune diseases (Braun *et al.*, 1983; Mantovani *et al.*, 1987; Gaspar *et al.*, 1988).

**Figure 5** The proliferative response to PHA (□) and anti-CD3 MAb (▨) of PBMNCs from STCC patients treated with intracavitary IFN-$\alpha_{2b}$ without evidence of tumour recurrence ($n = 14$) was significantly higher 3 and 6 months after finishing the treatment than that found before initiating it ($P < 0.01$). Results are expressed as the median and the range values of the different triplicate proliferation assays from the same group of subjects after 5 days of culture.

**Figure 6** There was no significant enhancement of the proliferative response of PBMNCs to (**a**) PHA and (**b**) anti-CD3 in patients with STCC of the bladder with evidence of tumour recurrence at 3 and 6 months after treatment with intravesical IFN-$\alpha_{2b}$ instillations. Results represent the mean individual values of the triplicate proliferation assays from each patient ($n = 3$) after 5 days of culture.

We have clearly demonstrated that there is a significant enhancement of the proliferative response of PBMNCs to polyclonal mitogens in the STCC patients who were treated prophylactically with intravesical instillations of IFN-$\alpha_{2b}$ and who remained free of tumour recurrence after 1 year of follow-up. This immunomodulator effect by the intravesical instillation of IFN-$\alpha_{2b}$ in STCC patients does not appear to be a consequence of any direct effect of this cytokine upon PBMNCs. It has been shown that intravesical instillation of IFN-$\alpha$ is associated with an immune infiltration of the bladder wall (P. Ferrari, personal communication). Thus, this systemic effect of intravesical IFN-$\alpha_{2b}$ could be explained by a local bladder wall immune activation with systemic functional consequences. It has been shown previously that the different cellular components of the immune system have multiple functional interactions as well as different distributions throughout the body (Goodman & LeFrançois, 1989: Picker *et al.*, 1990). Interestingly, the patients whose PBMNCs did not show an enhanced proliferative response to polyclonal mitogens after intravesical IFN-$\alpha_{2b}$ instillations were the ones whose disease recurred. This finding could be related to a different pattern of immune infiltrations on the bladder wall and/or to an abnormal immune system in this particular group of patients. Studies performed with intracavitary instillations of other immunomodulators such as BCG in the prophylactic treatment of patients with TCC have shown that the immunomodulators are associated with an increase in bladder wall infiltration by some cells and with a local production of cytokines (Peumchair *et al.*, 1989; Böhle *et al.*, 1990*a*,*b*).

IFN-$\alpha_{2b}$ also directly inhibits tumour cell proliferation (Fish *et al.*, 1983). Thus, this antiproliferative effect could also be involved in the mechanism of action of IFN-$\alpha_{2b}$ when given by intravesical instillations in the prophylactic treatment of patients with STCC of the bladder. However, IFN-$\alpha_{2b}$ also has an *in vitro* immunomodulator effect upon the cytotoxic immune effector cells (Carballido *et al.*, 1993). The present paper has demonstrated that intracavitary instillation of IFN-$\alpha_{2b}$ in patients with STCC of the bladder is also associated with an *in vivo* immunomodulator effect. Thus, it can be claimed that the immunomodulator effect of IFN-$\alpha$ is also implicated in the mechanism of action of this cytokine in the prophylaxis of tumour recurrences in patients with STCC. Further studies are needed to define the cellular and molecular mechanisms involved in this immunomodulator effect by intravesical IFN-$\alpha_{2b}$ instillations and their results might serve as clinical prognostic parameters for patients suffering from these malignancies.

The authors wish to thank C. Gonzalez for his expert technical help and C.F. Warren of the Instituto de Ciencias de la Educación of the UAH and J. Keller, MD, and B. Moragues, MD, for their linguistic assistance. This work was supported in part by a grant from Comunidad Autónoma de Madrid, SAL C265/91.

### References

ALVAREZ-MON, M., CASAS, J., LAGUNA, R., TORIBIO, M.L., ORTIZ-LANDAZURI, M. & DURANTEZ, A. (1986). Lymphokine induction of NK-like cytotoxicity in T cells from B-CLL. *Blood*, **67**, 228–232.

BÖHLE, A., GERDES, J., ULMER, A.J., HOSTETTER, A.G. & FLAD, H.D. (1990*a*). Effects of local bacillus Calmette–Guerin therapy in patients with bladder carcinoma on immunocompetent cells of the bladder wall. *J. Urol.*, **144**, 53–58.

BÖHLE, A., NOWC, CH., ULMER, A.J., MUSEHOLD, J., GERDES, J., HOFSTETTER, A.G. & FLAD, H.D. (1990*b*). Elevations of cytokines interleukin 1, interleukin 2 and tumor necrosis factor in the urine of patients after intravesical bacillus Calmette–Guerin immunotherapy. *J. Urol.*, **144**, 59–64.

BRAUN, D.P. & HARRIS, J.E. (1983). Serial immune functions testing to predict clinical disease relapse in patients with solid tumors. *Cancer Immunol. Immunother.*, **15**, 165–171.

BUBENICK, J., KIELER, J., TROMHOLT, V., HERMANN, G. & JANDLOVÀ, T. (1988). Defect in lectin-induced interleukin 2 production by peripheral blood lymphocytes of patients with invasive urinary bladder carcinoma. *Immunol. Lett.*, **18**, 115–118.

CARBALLIDO, J., ALVAREZ-MON, M., SOLOVERA, J., MENÉNDEZ-ONDINA, L. & DURANTEZ, A. (1990). Clinical significance of NK activity in patients with TCC of the bladder. *J. Urol.*, **143**, 29–33.

CARBALLIDO, J., MOLTÓ, L., MANZANO, L., OLIVIER, C., SLAMERÓN, O. & ALVAREZ-MON, M. (1993). Interferon alpha 2b enhances the natural killer activity of patients with transitional cell carcinoma of the bladder. *Cancer*, **72**, 1743–1748.

CRABTREE, G.R. (1989). Contingent genetic regulatory events in T lymphocyte activation. *Science*, **243**, 355-361.

DAVIS, L., VIDA, R. & LIPSKY, P.E. (1986). Regulation of human T lymphocyte mitogenesis by antibodies to CD3. *J. Immunol.*, **137**, 3758–3767.

FISH, E.N., BANERJEE, K. & STEBBING, N. (1983). Human leukocyte interferon subtypes have different antiproliferative and antiviral activities on human cells. *Biochem. Biophys. Res. Commun.*, **112**, 537–546.

GARCÍA-SUÁREZ, J., PRIETO, A., REYES, E., MANZANO, M., MERINO, J.L. & ALVAREZ-MON, M. (1993). Severe chronic autoimmune thrombocytopenic purpura is associated with an expansion of $CD56^{+}3^{-}$ natural killer cells subset. *Blood*, **82**, 1538–1545.

GASPAR, M.L., ALVAREZ-MON, M. & GUTIERREZ, C. (1988). Controversial role of interleukin 2 in inducing normalization of natural killer activity in systemic lupus erythematosus. *Clin. Immunol. Immunophatol.*, **49**, 204–214.

GLASHAN, R.W. (1990). A randomized controlled study of intravesical alpha-2b-interferon in carcinoma in situ of the bladder. *J. Urol.*, **144**, 658–661.

GOODMAN, T. & LEFRANÇOIS, L. (1989). Intraepithelial lymphocytes. Anatomical site, not T cell receptor form, dictates phenotype and function. *J. Exp. Med.*, **170**, 1569–1581.

GRIMM, E.A., MAZUMDER, A., ZHANG, H.Z. & ROSEMBERG, S.A. (1982). Lymphokine-activated killer cell phenomenon. Lysis of natural killer resistant fresh solid tumor cells by interleukin 2-activated autologous human peripheral blood lymphocytes. *J. Exp. Med.*, **155**, 1823–1841.

HISERODT, J.C. & CHAMBERS, W.C. (1988). Role of soluble cytotoxic factors in lymphokine-activated killer cell (LAK)-mediated cytotoxicity. *Ann. NY Acad. Sci.*, **532**, 395–404.

JURINICIC, C.D., ENGELMANN, U., GASCH, J. & KLIPPEL, K.F. (1988). Immunotherapy in bladder cancer with keyhole-limpet hemocyanin: a randomized study. *J. Urol.*, **139**, 723–726.

KEHRL, J.H., WAKEFIELD, L.M., ROBERTS, A.B., JAKOWLEN, S., ALVAREZ-MON, M., DERYNCK, R., SPORN, M.B. & FAUCI, A.S. (1986). The production of transforming growth factor beta by human lymphocytes and its potential role in regulation of T cell growth. *J. Exp. Med.*, **163**, 1037–1050.

LAMM, D.L., GRIFFITH, G., PETIT, L.L. & NSEYO, V.O. (1992). Current perspectives on diagnosis and treatment of superficial bladder cancer. *Urology*, **39**, 301–308.

LYNCH, C.F., PLATZ, C.E., JONES, M.P. & GAZZANIGA, J.M. (1991). Cancer registry problems in classifying bladder cancer. *J. Natl Cancer Inst.*, **83**, 429–433.

MANTOVANI, G., COIANA, A., MASSIDA, A., PROTO, E., FLORIS, C., MACCIO, A., PUSCEDDU, G. & DEL GIACCO, G.S. (1987). Interleukin 2 relationships with the cancer-related immunodeficiency: in vitro response to exogenous IL-2 by PHA-activated and non-PHA-activated peripheral blood mononuclear cells from cancer patients. *Diagn. Clin. Immunol.*, **5**, 104–111.

MELIEF, C.J.M. & KAST, W.M. (1991). T-cell immunotherapy of cancer. *Res. Immunol.*, **142**, 425–429.

MIZOGUCHI, H., O'SHEA, J.J., LONGO, D.L., LOEFFLER, C.M., MCVICAR, D.W. & OCHOA, A.C. (1992). Alterations in signal transduction molecules in T lymphocytes from tumor-bearing mice. *Science*, **258**, 1795–1798.

MORALES, A., EIDINGER, D. & BRUCE, A.W. (1976). Intracavitary Bacillus Calmette-Guerin in the treatment of superficial bladder tumors. *J. Urol.*, **116**, 180–183.

OLD, L.J. (1981). Cancer Immunology: the search for specificity. *Cancer Res.*, **41**, 361–375.

PEUMCHAIR, M., BENOIT, G., VIEILLEFOND, A., CHEVALIER, A., LEMAIGRE, G., MARTIN, E.D. & JARDIN, A. (1989). Analysis of mucosal bladder leukocyte subpopulations in patients treated with intravesical Bacillus Calmette–Guerin. *Urol. Res.*, **17**, 299–303.

PICKER, L.J., TERSTAPPEN, L.W.M.M., ROTT, L.S., STREETER, P.R., STEIN, H. & BUTCHER, E.C. (1990). Differential expression of homing-associated adhesion molecules by T cell subsets in man. *J. Immunol.*, **145**, 3247–3255.

RAGHAVAN, D., SHIPLEY, W.U., GARNICK, M.B., RUSSELL, P.J. & RICHIE, J.P. (1990). Biology and management of bladder cancer. *N. Engl. J. Med.*, **322**, 1129–1135.

RICHNER, J., AMBINDER, E.P., HOFFMAN, K., FEUER, E.J. & BEKESI, G. (1991). Number of helper T cells and phytohemagglutinin stimulation correlate in cancer patients. *Cancer Immunol. Immunother.*, **34**, 138–142.

RUSCETTI, F.W. (1990). Interleukin 2. In *Immunophysiology. The Role of Cells and Cytokines in Immunity and Inflammation*, Oppenheim, J.J. & Shevachs, E.M. (eds) pp. 46–66. Oxford University Press: Oxford.

SILVERBERG, E. & LUBERE, J.A. (1988). Cancer statistics. *Cancer*, **38**, 5–22.

TORTI, F.M. & LUM, B.L. (1987). Superficial bladder cancer. Risk of recurrence and potential role for interferon therapy. *Cancer*, **59**, 613–616.

URBAN, J.L., BURTON, R.C., HOLLAND, J.M., KRIPKE, M.L. & SCRIEBER, H. (1982). Mechanisms of syngeneic tumor rejection: susceptibility of host-selected progressor variants to various immunological effector cells. *J. Exp. Med.*, **155**, 557–573.

VALENTINE, M.A., TSOUKAS, C.D., RHODES, G., VAUGHAN, J.H. & CARSON, D.A. (1985). Phytohemagglutinin binds to the 20-kd molecule of the T3 complex. *Eur. J. Immunol.*, **15**, 851–854.

VAN DER BRUGGEN, P. & VAN DEN EYNDE, B. (1992). Molecular definition of tumor antigens recognized by T lymphocytes. *Curr. Opin. Immunol.*, **4**, 608–612.

WITJES, J.A. & DEBRUYNE, F.J.M. (1991). Optimal management of superficial bladder cancer. *Eur. J. Cancer*, **27**, 330–333.